# Exploring Outcome Measures for Exercise Intervention in People with Parkinson's Disease

**DOI:** 10.1155/2013/572134

**Published:** 2013-04-30

**Authors:** L. A. King, A. Salarian, M. Mancini, K. C. Priest, J. Nutt, A. Serdar, J. Wilhelm, J. Schlimgen, M. Smith, F. B. Horak

**Affiliations:** ^1^Department of Neurology, Oregon Health and Science University, Portland, OR 97239-3098, USA; ^2^Parkinson Center of Oregon, OP32, 3181 S.W. Sam Jackson Park Road, Portland, OR 97239-3098, USA; ^3^Department of Rehabilitation Services, Oregon Health and Science University, Portland, OR 97239-3098, USA

## Abstract

*Background*. It is widely believed that exercise improves mobility in people with Parkinson's disease (PD). However, it is difficult to determine whether a specific type of exercise is the most effective. The purpose of this study was to determine which outcome measures were sensitive to exercise intervention and to explore the effects of two different exercise programs for improving mobility in patients with PD. *Methods*. Participants were randomized into either the Agility Boot Camp (ABC) or treadmill training; 4x/week for 4 weeks. Outcome measures were grouped by the International Classification of Function/Disability (ICF). To determine the responsiveness to exercise, we calculated the standardized response means. *t*-tests were used to compare the relative benefits of each exercise program. *Results*. Four of five variables at the structure/function level changed after exercise: turn duration (*P* = 0.03), stride velocity (*P* = 0.001), peak arm speed (*P* = 0.001), and horizontal trunk ROM during gait (*P* = 0.02). Most measures improved similarly for both interventions. The only variable that detected a difference between groups was postural sway in ABC group (*F* = 4.95; *P* = 0.03). *Conclusion*. Outcome measures at ICF body structure/function level were most effective at detecting change after exercise and revealing differences in improvement between interventions.

## 1. Introduction

The progression of Parkinson's disease (PD) inevitably results in problems of balance, contributing to injuries, loss of mobility, increased health costs, and decline in quality of life. Delaying and minimizing these inevitable complications of PD with physical therapy exercise would have a major impact on patients and their families' quality of life, healthcare systems, and possibly even the course of disease progression.

Exercise has received much attention in the past decade as a way to delay the onset of mobility disability and there are a steadily increasing number of randomized controlled trials demonstrating that varying types of exercise improve some aspects of balance or gait [1–12]. However, it is difficult to determine whether a specific type of exercise program is more effective than another. One recurring obstacle, which makes it difficult to compare treatment approaches, is that studies use different types of outcome measures [13]. In addition, exercise studies almost always compare their favored type of intervention with a placebo or no intervention, rather than a head-to-head comparison of different types of exercise programs. The difficulty in comparing 2 potentially effective rehabilitation interventions is the need for very sensitive tests of change and a large number of subjects to differentiate exercise programs.

Although there is evidence from the animal literature that different exercise protocols result in different effects on synaptic and structural proteins in the brain, differentiating functional improvements in humans after different types of exercise has been more difficult to demonstrate [14]. For example, studies in rodents made Parkinsonian from 6-hydroxydopamine show that aerobic exercise on a treadmill results in increased angiogenesis whereas agility exercise that provides a mental challenge results in more synaptic plasticity [15]. However, a recent meta-analysis of human exercise studies could not distinguish relative benefits among the different types of exercise on clinical balance outcomes in patients with PD [16]. They found 15 randomized and quasi-randomized controlled trials of at least moderate quality in which the primary outcome measure was balance activity performance and found improved balance activity performance (Hedges' g, 0.33; 95% confidence interval, 0.11–0.55; *P* = 0.003). Although the pooled estimate of the effect of exercise was significant, the difference in effect size among different types of exercise intervention was not statistically significant (*P* = 0.166). A more recent Cochrane review that examined the effectiveness of physical therapy intervention for people with PD found similar results [17]. These authors found 33 trials with 1,518 participants that compared physical therapy with no intervention. They found evidence that most clinical measures improved after intervention (gait velocity, Timed Up and Go, Functional Reach, Berg Balance Scale, and Unified PD Rating Scale). However, they did not find any evidence of a difference in treatment effects across types of intervention. While no difference was noted between exercise interventions in both of these review papers, the outcome measures, except for gait velocity, were all clinical measures of performance or participation, which are often insensitive, subjective, and prone to ceiling effects, in contrast to objective measures of balance and gait [18, 19]. Therefore, exercise-specific differences in efficacy may have been overlooked. 

A recent review paper synthesized the effects of balance rehabilitation across the spectrum of PD disability [5]. This review systematically examined the impact of exercise interventions on balance outcomes on categories of disability defined by the World Health Organization in the International Classification of Functioning, Disability, and Health (ICF) model [20]. The ICF model categorizes outcomes according to 3 levels of human function: (1) “participation” includes problems an individual may experience in involvement in life situations (i.e., quality of life or falls) (2) “activity” is the execution of a task or action by an individual (i.e., performance during balance activities), and (3) “body structure and function” describes the physiologic function of various body systems (i.e., postural sway analysis). These authors found moderate evidence that exercise was effective for improving postural instability but only for the activity and body structure and function outcomes. They found limited evidence that exercise results in improvements in the participation level of function, such as quality of life measures or fall events.

While most clinicians believe that exercise improves function at all ICF levels in people with PD, there are many pressing questions remaining such as what are the most sensitive outcome measures at each ICF level for rehabilitation? What is the most effective type of exercise? For any of these questions to be satisfactorily and definitively addressed, it is critical that clinicians and researchers use sensitive outcome measures to adequately power a study and quantify change. 

The purpose of this study was (1) to determine which ICF outcome measures were most sensitive to exercise intervention and (2) to explore the effects of two different therapeutic exercise programs for improving mobility in PD. This exploratory aim will be used to determine the number of subjects needed to directly compare exercise programs in a future trial. To this end, we compared 2 published programs: (1) an Agility Boot Camp program (ABC) [21] versus (2) an aerobic treadmill (TT) approach [8]. Both exercise programs were customized and progressive and carried out at the same frequency and intensity by a physical therapist. Both exercise programs were selected to be efficacious for gait mobility, but only the ABC approach focused specifically on balance-related mobility. We hypothesized that objective measures of mobility at the body structure/function level would be more sensitive than clinical measures of mobility activity and participation to distinguish efficacy between the two approaches.

## 2. Methods

### 2.1. Design Overview

This study was a randomized, single-blinded intervention study for people with PD. Participants were randomized into one of two different exercise programs, ABC or TT. This trial was prospectively registered (NCT00982709) at http://clinicaltrials.gov/. 

All participants came into outpatient physical therapy at Oregon Health & Science University for two baseline pretest visits (four weeks apart) then participated in an intensive progressive exercise program under the direct supervision of a physical therapist, followed by a posttest visit after the completion of the exercise program. Insurance was billed for exercise visits and participants were partially compensated for testing to help offset copayments. The same outcome measures were collected at all three testing sessions by examiners blinded to group assignment. All pre- and posttesting sessions were performed in the same order and rest breaks were given as needed to avoid fatigue. All participants took their PD medication as normally indicated and were tested in the on state at the same time of day before and after intervention. 

### 2.2. Participants

Thirty-nine participants with idiopathic PD participated in the intervention with 20 randomized to ABC and 19 to TT intervention (Table 1). Participants were recruited from OHSU's Movement Disorders Clinic. Inclusion criteria included a diagnosis of idiopathic PD by a movement disorders neurologist, treatments with levodopa, between the ages of 45 and 85, and willing and able to come to the clinic 4 times per week for 4 weeks. Individuals were excluded from enrollment in the study if they were unable to ambulate unassisted, had other neurologic, cardiovascular, or orthopedic problems which could impact mobility, or had cognitive impairments that would limit participation in the intervention. Participants agreed not to alter their current medications or independent exercise habits during the study, as able. All participants signed informed consent forms approved by the Oregon Health & Science University Institutional Review Board. All work was conducted in accordance with the declaration of Helsinki (1964). 

### 2.3. Randomization and Intervention

The randomized group assignment was computer generated by an engineer in our laboratory, not associated with recruitment, study design, or implementation. All participants were assigned the same number of sessions (16 in total) for the same amount of time (75 minutes each session). These sessions took place four times per week for four consecutive weeks, under the direct instruction of one of four specially trained physical therapists. Fidelity of the intervention was maintained by unannounced observations and by having physical therapists document progression levels of training. Two of the physical therapists were trained in the ABC program and 2 were trained in the TT program. Analysis was done on intent-to-treat model.

### 2.4. Agility Boot Camp (ABC) Program

The theoretical basis for a novel, sensorimotor ABC exercise program is based on research from our laboratory and others that identified the primary neurophysiological constraints that limit balance and mobility in PD [21]. The exercises are designed as a circuit with 6 types of sports skill activities focused on improving basic postural systems: (1) pre-Pilates (2) kayaking to improve biomechanical constraints on joint flexibility, muscle strength, and postural alignment, (3) tai chi to improve kinesthesia and increase functional limits of stability, (4) boxing to improve anticipatory postural adjustments prior to stepping in multiple directions, (5) lunges to improve the speed and size of automatic stepping for postural correction, and (6) agility course to improve stability and coordination during gait challenged by quick changes in direction, avoiding or overcoming obstacles and simultaneously performing a secondary cognitive or motor task. 

Each activity was engaged for 10 minutes with rest periods and systematically progressed from beginning to intermediate to advanced levels by challenging (1) sensory integration (altering vision and/or surface conditions), (2) adding a secondary, cognitive task, (3) limiting external cues, and (3) increasing speed and resistance. Cool-down activities at the completion of the circuit included adapted floor Pilates: stretching of flexors and rotators, strengthening of extensors, and practice of transitional activities such as rising from a chair, getting onto the floor, rolling, and coming to stand from the floor [22]. This program and levels of progression are detailed in a previous publication [21].

### 2.5. Treadmill Training (TT) Program

The TT followed a previously published exercise protocol that demonstrated improved measures of gait, mobility, and quality in life in individuals with PD [8]. This program consisted of fast walking on a treadmill for up to 30–45 min as tolerated per session, with an additional 10 minutes of warm-up and cool-down adapted Pilates, that was the same as the ABC arm. Treadmill intensity was started at 80% of each participant's natural, overground, gait velocity and was increased to 90% after a week. Natural gait velocity was measured at the beginning of each week with a stopwatch prior to each treadmill training by asking participants to walk 25 feet. From the third week of training, the treadmill speed was gradually increased to reach a goal of 5% to 10% above that week's overground walking speed. Participants were allowed to hold onto the railing to focus on gait training. Therapists encouraged participants to increase stride length and height and to keep their upper body erect during the training period but were not allowed to work with the patient on any direct aspects of balance, beyond that used for walking on a treadmill. Safety harnesses were worn at the discretion of the physical therapist and none of the participants used a body weight support harness. 

All participants were instructed to take their anti-PD medications as normal during the exercise program. All participants were instructed not to change their anti-PD medications during the study if possible.

### 2.6. Outcome Measures

Outcome measures were classified at 3 levels (participation, activity, and body structure and function) according to the International Classification of Function and Disability (ICF) model. 

#### 2.6.1. Level 1 of ICF: Participation

To assess changes at the participation level, we used the Parkinson's Disease Questionnaire (PDQ-39) [23] for quality of life, the Activities of Balance Confidence Scale (ABC) [24], and the Activities of Daily Living from the Unified Parkinson's Disease Rating Scale (UPDRS Part II) [25]. The PDQ-39 is a frequently used, validated questionnaire that was designed as a tool for determining treatment effect on eight different domains in individuals with PD: mobility, activities of daily living, emotional well-being, stigma, social support, cognition, communication, and bodily discomfort. Each of the eight sections has a separate total score, on a scale of 0 (perfect health) to a maximum score of 100% (worse health). This scale has been found to be a valid and reliable way in which to measure quality of life for individuals with PD [26, 27]. The ABC is a reliable method for detecting loss of balance confidence in an aging population and for those with PD [28]. The test is comprised of sixteen questions that are averaged for one total score. A score of less than 68% indicates low mobility [24]. The UPDRS Activities of Daily Living is a 13-item questionnaire focusing on the effects of PD on activities of daily living [25].

#### 2.6.2. Level 2 of ICF: Activity

To characterize changes at the activity level, we measured overall changes in balance, mobility function, and disease severity using the Mini-BESTest, Berg Balance Scale, and the UPDRS Part III. The Mini-BESTest test is a 14-item test that focuses on dynamic balance; specifically, anticipatory transitions, postural responses, sensory orientation, and dynamic gait. Each item is scored from 0 to 2; a score of 0 indicates that a person is unable to perform the task while a score of 2 is normal. The highest score, indicating no impairment, is 28 [29]. Berg Balance Scale is a 14-item clinical test that measures balance by assessing performance on specific functional tasks [30]. Each task is scored from 0 to 4 (0: unable to 4: normal), for a maximum best score of 56 points. Scoring is based on criterion that is specific to each task. The UPDRS III [25] is a motor examination used to assess disease severity. This test has a maximum score of 108; each item is scored from 0 (not affected) to 4 (most severely affected). These clinical scales were categorized at the “activity” level because though they measure subcomponents on the impairment level (i.e., tremor, rigidity), it is not a physiologic assessment and the overall score is based on the execution of a task or action by an individual.

#### 2.6.3. Level 3 of ICF: Body Structure and Function

To characterize body structure and function, we objectively measured gait and turning using the Instrumented Timed Up and Go (ITUG) [31] and balance during quiet standing with the Instrumented Sway (ISway) [32]. The participants wore a portable data-receiver (X-Bus) connected with wires to 6 MTX XSens sensors (49A33G15, XSens, Enschede, The Netherlands) positioned on (1) the posterior trunk at the level of L5, near the body center of mass, (2) one on the anterior shank of each leg, (3) one on the dorsum side of each arm, and (4) the sternum. The sensor recorded 3D accelerations and angular velocity while the controller wirelessly streamed data to a laptop via Bluetooth. A custom MATLAB [33] graphical interface was used to acquire and store data. Later, data were automatically analyzed using Mobility Lab software (APDM Inc., Portland, OR, USA) [34].

The ITUG involved instructing participants to stand up, walk over a tape on the ground 7 meters away, turn around, walk back, and sit down. ITUG uses automatic analysis algorithms from APDM's Mobility Lab to objectively calculate temporal and spatial gait metrics during straight ahead gait as well as turning metrics. In the present study, we calculated the median of 3 ITUG trials of the following metrics: stride velocity, peak arm speed, horizontal trunk range of motion, and turn duration [31]. These measures were chosen because previous studies suggest that they are sensitive to early PD [31] and they comprehensively characterize commonly impaired aspects of PD (slowed gait, slowed turns, decreased arm swing, and decreased trunk rotation during gait).

The ISway [32] involved instructing participants to stand with arms at their sides and looking straight ahead at an art poster for 30 seconds across 3 trials. The size of participants' stance was fixed and made consistent with a spacer block momentarily placed between the feet. ISway uses automatic analysis algorithms from APDM's Mobility Lab to objectively calculate amplitude, velocity, and frequency of sway during quiet standing. In the present study, we calculated the median of 3 ISway trials for the bidirectional range of sway.

### 2.7. Statistical Analysis

Subject characteristics between the two exercise groups were summarized with group means and compared with *t*-test to investigate any group differences. Stability of outcome measures during the two baseline measures 4 weeks apart was compared using intraclass correlation (ICC type (1, 1)). To determine the most sensitive outcome measures after exercise intervention, we calculated the standardized response mean (SRM) for each measure as well as paired *t*-tests to determine if statistical change occurred before and after intervention. SRM is the mean change (d) reported in units of standard deviation of change (SD_diff_), SRM = d/SD_diff_. For SRM, a value of 0.20 represents a small change, of 0.50 a moderate, and 0.80 represents a large change [35]. To compare the effects of the ABC versus the TT intervention, we used *t*-tests to evaluate the benefit of exercise (post minus pre exercise value) between the 2 programs. To determine the necessary number of subjects needed to distinguish benefits from the different exercise programs, we used the “pwr” package in R program for a power and sample size analysis. For power analysis a significance level of *α* = 0.05 and a power = 1 − *β* = 0.80 were assumed. 

## 3. Results

**Figure 1:**
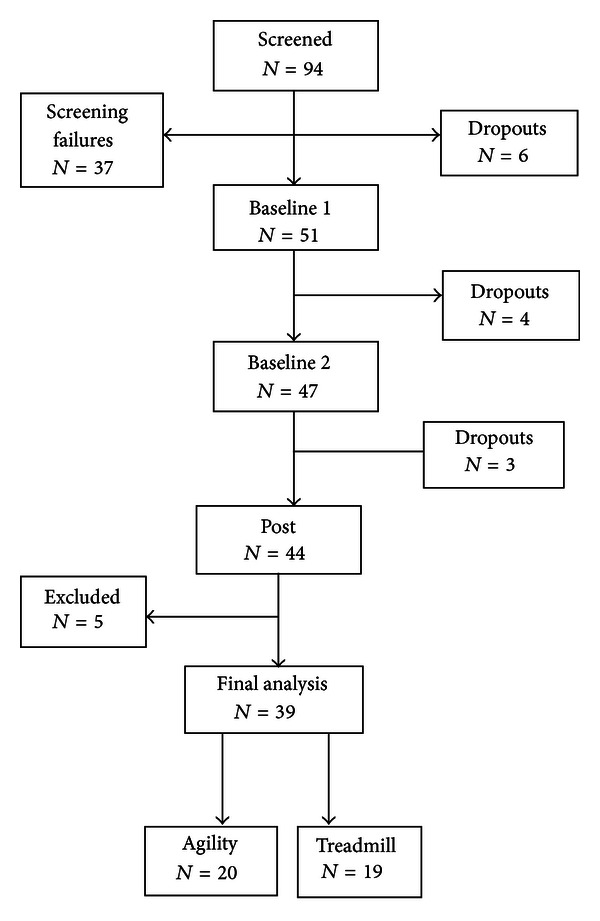
Consort diagram of recruitment and participation.

Of the 94 people recruited for the study, 37 did not meet inclusion criteria and were not scheduled for a study visit (Figure 1). Six people dropped out before the study began for reasons of convenience, distance to travel, or costs associated with participation. Seven more people dropped out during the course of the study for similar reasons. At the end of the study, 44 people completed the study. Five of these people were excluded from the analysis for the following reasons: one person was subsequently diagnosed with multiple systems atrophy with significant cognitive decline, 2 people had surgery during the course of the study, 1 person was unable to ambulate without her walker during the pretest, and 1 person had a medication change during the course of the study. The target number of visits for this intervention was 16 exercise sessions over the course of four weeks. However, due to the logistics of coordinating with rehabilitation services, standard-of-care, and insurance policies, the average number of intervention visits prior to posttesting was 16 ± 1 visits.

 Adverse events: no soreness or musculoskeletal complaints were reported. One participant fell during lateral stepping during an ABC training session but no injury occurred. No other adverse events were reported. 

Baseline characteristics of the participants are shown in Table 1. No statistical differences were found in baseline characteristics between the 2 intervention groups.

## 4. Stability of Outcome Measures over 4 Weeks without Intervention

All of the outcome measures were stable prior to intervention. The ICCs between two baseline measures taken 4 weeks apart were all above 0.7 and there was no significant difference between baseline measures.  Table 2 provides ICC values for each outcome measure for the 2 baseline tests. Each section of PDQ39 is detailed in Table 4.

## 5. Responsiveness of Outcome Measures to Exercise

To determine which measures were most responsive to intense exercise intervention, we initially grouped all subjects together, regardless of exercise intervention assignment, to obtain a higher power. We found that 4 out of 5 measures in the body structure and function ICF level improved, 2 of 3 measures in the activity level improved, and just 1 of 3 measures in the participation level improved after 4 weeks of exercise (Table 3).

## 6. Which Measures Distinguish between ABC and TT Intervention?

**Figure 2:**
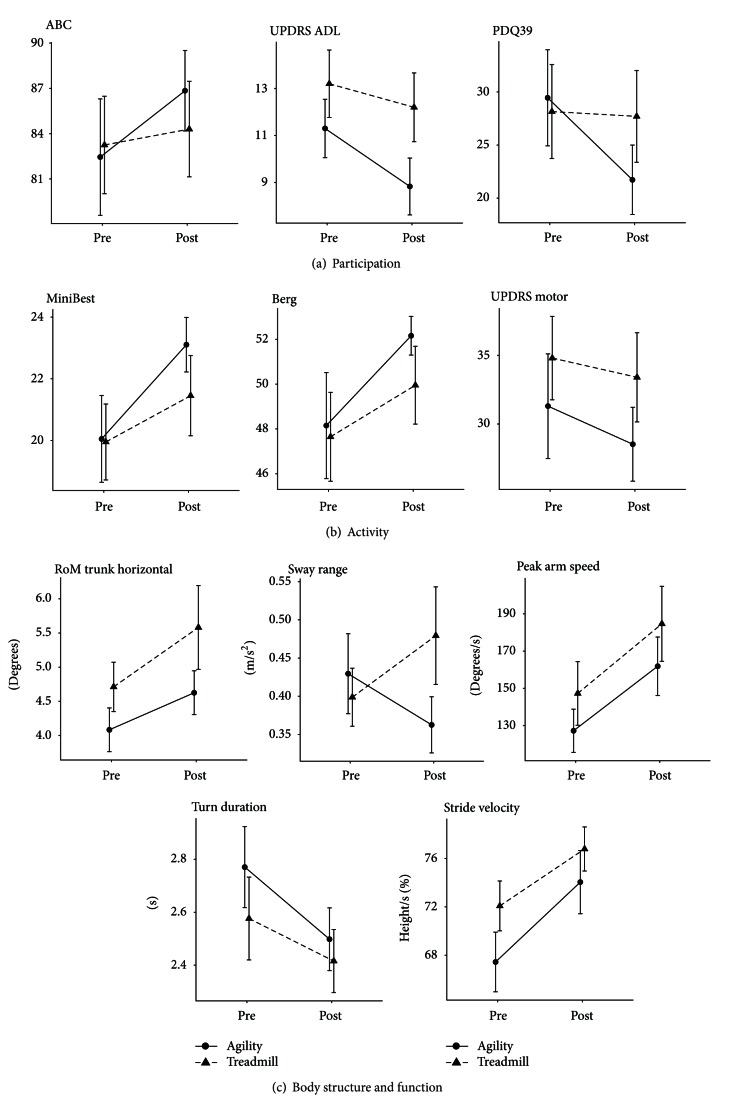
Pre- to Postintervention plots for each exercise group, Agility Boot Camp and treadmill intervention, for each variable according to International Classification of Function and Disability.

Both groups improved in numerous measures similarly but only one measure, at the body structure and function ICF level (sway range, Figure 2), showed a significant interaction effect for the different outcomes of the exercise programs (*F* = 4.95; *P* = 0.03).

Though this study was not powered to detect a difference in outcomes between the two short exercise intervention programs, we saw trends suggesting that the ABC program may result in greater improvement for many outcomes (Figure 2). *t*-tests were performed on the groups separately. The ABC group significantly improved in balance (Mini-BESTest *t* = − 3.1; *P* = 0.007), gait (stride velocity *t* = − 2.27; *P* = 0.04), peak arm speed (*t* = − 3.31; *P* = 0.004), and ROM of trunk during gait (*t* = − 2.48; *P* = 0.02). The TT group improved in balance (Mini-BESTest *t* = − 2.7; *P* = 0.01), gait (stride velocity *t* = − 2.97; *P* = 0.01), and peak arm speed (*t* = − 3.25; *P* = 0.005). 

### 6.1. Power Analysis Depends on Outcome Measure

In our study, the 3 outcome measures that showed the most promising ability to detect a change between the ABC and the TT program were (1) range of postural sway, (2) the Mini-BESTest, and (3) ROM of the trunk during gait. A power analysis revealed that to detect program differences, one would need 33 people when using sway as an outcome measure for balance, 273 people when using ROM of the trunk during gait, and 283 people if using the Mini-BESTest. All other outcome measures required greater than 1000 people to detect a programmatic difference due to small effect sizes. 

## 7. Discussion

The main finding of this study is that improvement after exercise intervention was most readily measured at the body structure and function level of the ICF. Only sway range during quiet stance differentiated outcomes for the 2 short-term, high-frequency mobility intervention programs. Despite different rehabilitation focus, aimed at either dynamic balance for agility (ABC intervention) or aerobic treadmill training (TT intervention), people with PD showed similar improvements on many clinical measures. 

The importance of sensitive, specific outcome measures for clinical trials and clinical practice cannot be underestimated. Though this study was underpowered to see differences between programs with a direct comparison, our results showed trends suggesting that a greater number of outcome measures improved after ABC exercise than treadmill. The only measures with an interaction effect was postural sway which decreased after ABC exercise. Postural sway is considered a sensitive measure of balance and though increased sway is associated with falls in the literature, it is not a measure typically assessed in the clinic. The findings from our study suggest that an important reason for a lack of obvious difference between different types of rehabilitation program effectiveness, both in our study and in the literature, may be due to insensitive outcome measures, underpowered studies, or both.

Most exercise intervention studies do not find differences in outcomes when comparing two potentially efficacious physical therapy interventions. For example, both the SCILTS and the LEAPS trials found that conventional, overground walking in rehabilitation of people with spinal cord injury (SCILT) and conventional, home-based exercise for people with stroke (LEAPS) produced similar results compared to people using advanced technology involving the bodyweight supported treadmill training [36, 37]. Dobkin and Duncan [38] published a review of robotic-assisted stepping devices and bodyweight-supported treadmill and revealed little solid evidence that such approaches are more beneficial than standard of care in physical therapy [38]. The primary outcome measures for these studies were primarily standard noninstrumented scales used in the clinic such as the Berg Balance Scale, Functional Independence Measures, and overground walking speed with a stopwatch. These clinical measures may not be sensitive enough to detect differences between types of intervention. Further analysis revealed that more than 1000 people would be needed to possibly show a difference between the interventions in the SCILT trial [38]. 

Also of note is that the UPDRS did not change in either our ABC or TT group after exercise. While this was not surprising since one would not expect 4 weeks of exercise to significantly change many motor signs measured in the UPDRS (hand movements, tremor, speech, and facial expression), it is important since many studies of exercise intervention for PD use the UPDRS as an outcome measure [2, 6, 7]. However, a very long duration, 12-month, 2 times per week agility intervention based on therapeutic tango lessons, showed a significant improvement in the UPDRS in the off state [39]. The on levodopa state was not tested. 

The main focus of the ABC program was practicing to control body center of mass during a variety of tasks with a moving base of support and under a variety of sensory and cognitive challenges. For example, the ABC group practiced lunges with big steps and progressed to lunging without visual feedback or while performing a dual task. This group did not specifically practice walking or sway during quiet stance. Therefore, our results suggest that the participants in the ABC group were able to control their center of mass more efficiently; even in conditions they did not practice, indicating a carryover of skills to new tasks. Similarly, the TT group improved in balance scores that were not specifically practiced during gait training. It can be argued, however, that walking on a treadmill, especially without use of handrails as they progressed, required balance control so this group may have had dynamic balance training as well. The results may represent a specificity of training effect since the ABC program was designed to target underlying impairments, perhaps similar to what is tested at the function/structure level. Similarly, perhaps there was no difference between groups at the activity level because both groups addressed some aspects of balance and gait leading to improvements in these areas across both groups. 

One limitation of this study is that the study design prevented us from calculating the minimal detectable change for our measurements. The time between repeated measures before intervention was too long to reliably measure the minimal detectable change. This information would be valuable in future studies, particularly for the new, instrumented measures that have not been documented. Though the exercise sessions were frequent and for longer duration per session than traditional physical therapy sessions, the overall timeline of exercise intervention was relatively short (4 weeks). The design of our study included people who were coming in for therapy, billing to insurance. This may have biased our sample since people without insurance or certain types of insurance could not participate. Similarly, our participants came in for exercise 4 times per week for 75 min, precluding those people with very poor endurance or who had difficulty leaving their homes to travel to the clinic. This frequency was chosen in the design in order to maximize improvements in a short duration since we were unable to carry out a long-term study. Our participants were relatively of high-level functioning so it may be more difficult to detect change after exercise compared to people with less mobility. Because each person participating had to pay a copayment each visit, it was an expensive way to administer an intense exercise program. Future studies should investigate less expensive group classes.

## 8. Conclusions

This study suggests that future randomized clinical trials of mobility intervention should include objective measures of balance and gait at the body structure and function level of the ICF to distinguish between two types of physical therapy intervention with reasonable size groups. Physical therapists in the clinic, however, should consider using responsive, objective outcomes at each of the ICF levels to help direct their intervention and document change. Outcome measures should be used strategically to help therapists understand what is and what is not changing after intervention and appropriate outcomes with frequent measures can help guide therapeutic intervention.

## Figures and Tables

**Table 1:** Participant characteristics.

Variables	Intervention groups		*P* value
	ABC	TT	
Age (yr)	65.7 ± 8.3	65.1 ± 7.3	0.70
Height (cm)	172.1 ± 8.0	175.0 ± 10.5	0.97
Weight (kg)	75.5 ± 16.3	81.8 ± 16.2	1.3
Gender	8 (F); 12 (M)	6 (F); 13 (M)	NA
UPDRS motor	33.4 ± 16.6	32.3 ± 13.8	0.72
H&Y	2.5 ± 0.8	2.4 ± 0.6	0.23

**Table 2:** Baseline stability of outcome measures.

Variables	ICC	Real difference ± SD
Participation		
PDQ-39	0.794	−4.6 ± 13.8
ABC	0.767	4.2 ± 11.3
UPDRS-ADL	0.914	−0.3 ± 2.6
Activity		
Mini-BESTest	0.879	0.9 ± 3.2
Berg Balance Scale	0.951	−0.3 ± 3.0
UPDRS motor	0.917	−0.9 ± 6.1
Body structure and function		
Turn duration	0.827	−0.1 ± 0.4
Stride velocity	0.886	0.03 ± 5.1
Peak arm speed	0.802	4.5 ± 40.1
ROM trunk horizontal	0.889	−0.2 ± 0.9
Sway range	0.695	−0.01 ± 0.17

**Table 3:** The effects of exercise intervention for each category of ICF.

Variables	Pre- mean (SD)	Post- mean (SD)	SRM	*P* value
	Participation			

PDQ-39	28.8 ± 19.8	24.9 ± 17.0	−0.331	0.127
ABC	82.9 ± 15.7	85.5 ± 12.9	0.319	0.152
UPDRS-ADL	12.3 ± 6.00	10.6 ± 6.07	−0.531	0.021*

	Activity			

Mini-BESTest	20.0 ± 5.83	22.3 ± 4.97	0.814	0.001***
Berg Balance Scale	47.9 ± 9.64	51.0 ± 6.17	0.649	0.004**
UPDRS motor	33.1 ± 15.3	31.1 ± 13.3	−0.304	0.255

	Body structure and function			

Turn duration	2.68 ± 0.66	2.46 ± 0.50	−0.609	0.030*
Stride velocity	69.6 ± 9.88	75.3 ± 9.71	0.772	0.001***
Peak Arm Speed	137 ± 60.7	173 ± 75.4	0.986	0.001***
ROM trunk horizontal	4.38 ± 1.45	5.07 ± 2.04	0.523	0.019**
Sway range	0.41 ± 0.19	0.42 ± 0.23	0.051	0.406

*Significance at the 0.05 level.

**Significance at the 0.01 level.

***Significance at the 0.001 level.

**Table 4:** Effects of exercise on the PDQ-39 subsections.

Variables	Pre- mean (SD)	Post- mean (SD)	SRM	*P* value
PDQ-39 total	28.8 ± 19.8	24.9 ± 17.0	−0.33	0.127
PDQ mobility	8.05 ± 8.06	6.31 ± 6.47	−0.37	**0.09**
PDQ ADL	5.28 ± 4.44	3.92 ± 3.77	−0.49	0.01**
PDQ emotional	4.18 ± 3.54	4.13 ± 3.53	−0.02	0.85
PDQ stigma	2.23 ± 2.33	2.08 ± 2.15	−0.05	0.72
PDQ social	0.88 ± 1.14	0.87 ± 1.17	−0.01	0.44
PDQ cognitive	2.88 ± 2.37	2.71 ± 2.13	−0.09	0.78
PDQ communication	2.30 ± 2.15	2.03 ± 1.90	−0.15	0.49
PDQ discomfort	3.03 ± 2.27	2.82 ± 2.12	−0.12	0.51

**Significance at the 0.01 level.
